# Enzymatically-mineralized double-network hydrogels with ultrahigh mechanical strength, toughness, and stiffness

**DOI:** 10.7150/thno.77417

**Published:** 2023-01-01

**Authors:** Li Wang, Wei Zhao, Yining Zhao, Wei Li, Guodong Wang, Qiang Zhang

**Affiliations:** 1 Department of Stomatology, Changzheng Hospital, Naval Medical University, Shanghai, 200003, P. R. China.; 2 Shanghai Key Laboratory of Regulatory Biology, School of Life Sciences, East China Normal University, Shanghai, 200241, P.R. China.

**Keywords:** hybrid hydrogel, double network, enzymatic mineralization, ultrahigh mechanical properties, subchondral bone defect repair

## Abstract

**Background:** Synthetic hydrogels are commonly mechanically weak which limits the scope of their applications.

**Methods:** In this study, we synthesized an organic-inorganic hybrid hydrogel with ultrahigh strength, stiffness, and toughness via enzyme-induced mineralization of calcium phosphate in a double network of bacterial cellulose nanofibers and alginate-Ca^2+^.

**Results:** Cellulose nanofibers formed the first rigid network via hydrogen binding and templated the deposition of calcium phosphate, while alginate-Ca^2+^ formed the second energy-dissipating network via ionic interaction. The two networks created a brick-mortar-like structure, in which the “tortuous fracture path” mechanism by breaking the interlaced calcium phosphate-coated bacterial cellulose nanofibers and the hysteresis by unzipping the ionic alginate-Ca^2+^ network made a great contribution to the mechanical properties of the hydrogels.

**Conclusion:** The optimized hydrogel exhibited ultrahigh fracture stress of 48 MPa, Young's modulus of 1329 MPa, and fracture energy of 3013 J/m^2^, which are barely possessed by the reported synthetic hydrogels. Finally, the hydrogel represented potential use in subchondral bone defect repair in an *ex vivo* model.

## Introduction

Hydrogels have found wide applications in biomedical areas, such as drug delivery 1, tissue engineering 2, biosensors 3, and structural implants 4, owing to their nature of high-water content and three-dimensional network and their similarity to the extracellular matrix 5. For practical uses, hydrogels with variable mechanical properties of strength, stiffness, toughness, and/or elongation are required for different application scenarios 6-8. However, most synthetic hydrogels are mechanically weak 9. They generally have low strength of ~100 kPa, stiffness of ~10 kPa, and toughness of ~10 J/m^-2^ 10. In comparison, the natural load-bearing tissues like cartilage, tendons, skins, and muscles have high water contents of ~70% but are still mechanically strong and durable 11. For instance, cartilages have a tensile strength of up to 25 MPa, Yong's modulus larger than 10 MPa, and fracture energy of around 1000 J/m^-2^ 12, and tendons possess a tensile strength of ~25 MPa and Yong's modulus of ~1.2 GPa 13. Therefore, the weak mechanical properties of synthetic hydrogels severely limit their applications in biomedical areas.

In the past two decades, great efforts have been devoted to synthesizing hydrogels with improved mechanical properties. The tough hydrogels have been well made by introducing energy dissipation mechanisms that are majorly accessed via constructing a double network 14-17. A typical case is that the hydrogels created by covalently and ionically crosslinked double networks achieve giant fracture energy of ~9000 J/cm^2^ 18. However, the ultrahigh toughness is a compromised result of low strength and stiffness (156 kPa for fracture stress, and 29 kPa for Young's modulus) 18. Historically, stiffness and toughness are considered mutually exclusive in a hydrogel, and thus the synthesis of hydrogels with both high stiffness and toughness is not easy. To stiffen hydrogels, diverse strategies, such as increasing solid contents and/or crosslink densities 19-21, forming crystallite regions 22-23, introducing hydrogen bonding 17, creating conjoined networks 24, adding hydrophobic interaction 25, and integrating macroscale fibers 26,27, are extensively exploited based on the double network structures. As a result, the stiffness of the hydrogels that possess high toughness has been well improved. In most cases, the elastic moduli of the tough hydrogels are enhanced up to 50 MPa 16,23,28. Biomineralization is the major process in organisms to form ultra-stiff and tough tissues like bone and teeth 29, in which the organic components form a three-dimensional matrix and template the nucleation and growth of inorganics. Similarly, the organic-inorganic hybrid construction made via filling inorganic components and biomineralization has also been utilized to stiffen and strengthen hydrogels 30-32. For instance, enzyme-induced calcium deposition in a polymer matrix is recently developed to prepare ultra-stiff and tough hydrogels 33. The hybrid hydrogels represent ultrahigh Young's moduli up to 440 MPa and fracture energies larger than 1300 J/m^2^. Except for stiffness and toughness, strength is also a concern for synthetic hydrogels since certain natural tissues are ultra-strong like cartilages representing high strength of ~25 MPa 12. However, few synthetic hydrogels can simultaneously possess ultrahigh strength, stiffness, and toughness. Even the mineralized hydrogels have ultrahigh stiffness and toughness, their tensile strength however is still relatively low (~1 MPa) 33.

In this study, we developed a new strategy to synthesize hybrid hydrogels with ultrahigh strength, stiffness, and toughness. In the method, a double network consisting of bacterial cellulose (BC) nanofibers and Ca^2+^-crosslinked alginate (alginate-Ca^2+^) was constructed, and then enzyme-induced calcium phosphate (CaP) mineralization occurred in the gel matrix. BC nanofibers acted as a stiff-reinforced component to create the first rigid network via hydrogen binding, and alginate-Ca^2+^ formed the second energy-dissipating network via ionic interaction. BC nanofibers also templated CaP deposition to form CaP-coated BC (BC@CaP). BC@CaP and alginate-Ca^2+^ intertwined in the hydrogels to form a brick-and-mortar-like structure. When hydrogel stretched, the “tortuous fracture path” mechanism by breaking interlaced BC@CaP nanostructures and the hysteresis by unzipping alginate-Ca^2+^networks made a great contribution to the mechanical properties, resulting in highly strong, stiff, and tough hydrogels. Finally, the hybrid hydrogel was well used for subchondral bone defect repair.

## Results and Discussion

### Fabrication of the organic-inorganic hybrid hydrogels

**Figure 1:**
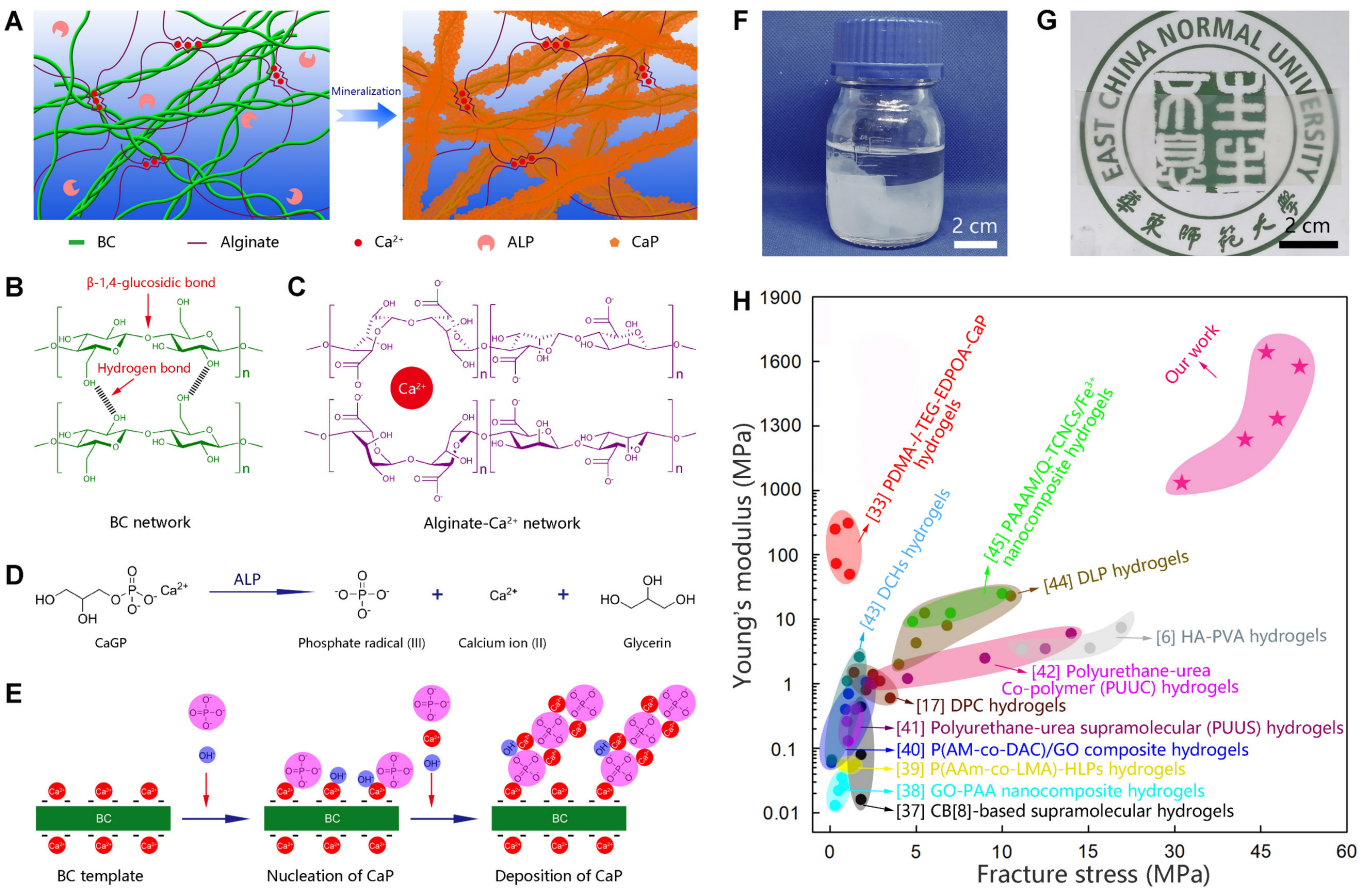
** Enzyme catalyzed mineralization in BC/alginate-Ca^2+^ double-network hydrogel.** (**A**) Schematic diagram of the mineralization process. (**B and C**) BC and alginate-Ca^2+^ formed the double-network hydrogel. (**D**) ALPs catalyze the transformation of CaGP into Ca^2+^, PO_4_^3-^, and glycerin. (**E**) Ca^2+^ and PO_4_^3-^ deposit on the surface of BC nanofibers to form CaP nanostructures. (**F**) B_80_A_20_ hydrogel mineralized in 0.20 M TEA buffer containing 5 g/L of CaGP. (**G**) Photograph of B_80_A_20_-M_6_ hydrogel. (**H**) Ashby plot shows Young's modulus vs. fracture stress of B_80_A_20_-M_6_ hydrogel and other stiff and/or tough hydrogels reported in the literature.

Biomineralization is the major process in organisms to form ultra-stiff and tough tissues like bone and teeth, in which the organic components form a three-dimensional matrix and serve as a template to control the nucleation and growth of inorganic components 29. In this case, we prepared the hybrid hydrogels, that is B_x_A_100-x_-M_y_ hydrogels (B stands for BC, A does for alginate-Ca^2+^, and M does for mineralization; x means the weight percentage of BC in the dry film of BC and alginate, and y means the mineralization days), via enzyme-catalyzed mineralization of CaP in a double network of BC and alginate-Ca^2+^ (Figure 1A). The organic matrix was first prepared by mixing BC and alginate via a blade-casting method 34. BC was used as the major component in the organic matrix due to its excellent mechanical properties of ultrahigh tensile stress and elastic modulus (Figure 1B) 35. The alginate-Ca^2+^ network was introduced to enhance the toughness of hydrogels via an energy-dissipating mechanism (Figure 1C). Alkaline phosphatases (ALPs) were added to the matrix to catalyze the deposition of CaP 36. The matrix was then immersed in a triethanolamine (TEA) buffer (0.2 M, pH=9.8) containing calcium glycerophosphate (CaGP, 5 g/L). ALPs catalyzed the decomposition of CaGP (Figure 1D). BC nanofibers had a negatively-charged surface (Figure S1), which absorbed Ca^2+^ and then templated the nucleation and deposition of CaP (Figure 1E). B_80_A_20_ hydrogel loaded with ALPs was immersed in TEA buffer for mineralization. However, the buffer solution became cloudy in 1-2 h (Figure S2A), because ALPs were too small (2.8 nm) to be trapped in the gel matrix 33. To prevent enzyme leaking, ALPs were further crosslinked by different amounts of poly-glutaraldehyde (PGL) to form large-size PGL/ALP nanoparticles 33. The enzyme activities of ALPs in PGL/ALP nanoparticles were further determined according to the calibration curve of p-nitrophenol, as ALPs catalyzed the decomposition of p-nitrophenol phosphonate to release p-nitrophenol. (Figure S3). The hydrodynamic sizes of PGL/ALP nanoparticles were increased along with the amounts of PGL, but the enzyme activities were gradually reduced (Figure S4 and S5). Finally, PGL/ALP nanoparticles at a weight ratio of ALP: PGL = 10: 1 were chosen for the following study due to that the nanoparticles had a relatively large size of 108 nm and also maintained 85% of the native enzyme activity (4.94 U/mg, Figure S4 and S5). As a result, B_80_A_20_ hydrogel loaded with PGL/ALP nanoparticles could well maintain the solution clear (Figure S2B). Even after mineralization for 6 days, the solution was still clear (Figure 1F), and a transparent mineralized B_80_A_20_-M_6_ hydrogel was obtained (Figure 1G). We compared the mechanical properties of our hydrogels with these of the strong hydrogels reported in the literature 6,17,33,37-45. As shown in Figure 1H, our hydrogels' fracture stresses and Young's moduli were much larger than these of the reported hydrogels. The largest fracture stress of our hydrogels was 51.8±1.4 MPa and the largest Young's modulus was 1640.0±150.0 MPa (Figure 1H).

### Mechanical properties of the hybrid hydrogels

**Figure 2:**
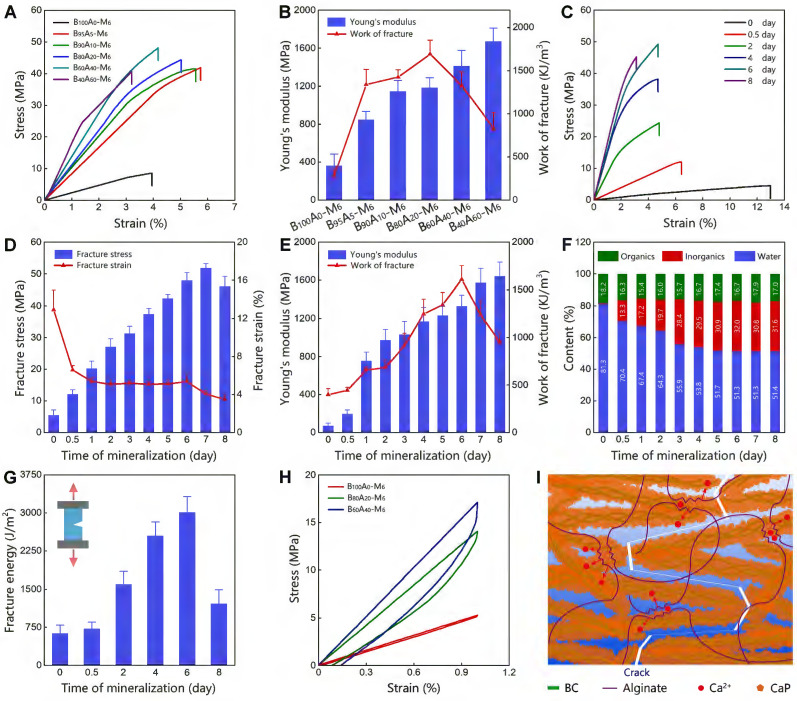
** Mechanical properties of mineralized hydrogels.** (**A**) The stress-strain curves of B_x_A_100-x_-M_6_ hydrogels containing different proportions of BC and alginate. (**B**) The Young's modulus and work of fracture for B_x_A_100-x_-M_6_ hydrogels (n=5). (**C**) The stress-strain curves of B_80_A_20_-M_y_ hydrogels mineralized for different days. (**D**) The fracture stress and fracture strain of B_80_A_20_-M_y_ hydrogels (n=5). (**E**) The Young's modulus and work of fracture of B_80_A_20_-M_y_ hydrogels (n=5). (**F**) The component proportions of B_80_A_20_-M_y_ hydrogels (n=5). (**G**) The fracture energies of B_80_A_20_-M_y_ hydrogels (n=5). (**H**) The loading-unloading curves of B_100_A_0_-M_6_, B_80_A_20_-M_6_, and B_60_A_40_-M_6_ hydrogels. (**I**) Schematic diagram shows the “tortuous fracture path” among BC@CaP nanostructures and the hysteresis of unzipping alginate/Ca^2+^networks when hydrogel stretched.

Initially, we employed BC as the exclusive component for the organic matrix. The as-obtained B_100_A_0_-M_6_ hydrogel represented Young's modulus of 360.8±125.7 MPa and work of fracture of 279.0±88.0 kJ/m^3^ (Figure 2A-B), which was highly stiff and tough but did not stand out compared with the mineralized polymer hydrogels 33. Consequently, we incorporated BC with alginate-Ca^2+^ at different weight ratios to form double-network hydrogels (Figure S6), in which alginate-Ca^2+^ was used for dissipating energy via breaking the ionic crosslinking of guluronate blocks 18. The proportions of alginate were tuned in a range of 0-60 wt% (dried content of B_x_A_100-x_ hydrogels), and the mechanical properties of the hydrogels were measured. Among them, B_80_A_20_ hydrogels showed both relatively high Young's modulus (212.8±43.3 MPa) and work of fracture (386.1±119.3 KJ/m^3^, Figure S7 and S8). Furthermore, B_x_A_100-x_ hydrogels containing different amounts of alginate were mineralized for 6 days. The mineralized hydrogels of B_90_A_10_-M_6_ and B_80_A_20_-M_6_ were quite transparent, while the other ones were cloudy (Figure S9), implying CaP nanocomposites formed in the two hydrogels of B_90_A_10_-M_6_ and B_80_A_20_-M_6_ were small 33. The mechanical properties of B_100_A_100-x_-M_6_ hydrogels were further determined by testing the stress-strain curves. The fracture stresses were remarkably enhanced in the hydrogels containing alginate-Ca^2+^ (Figure 2A and S10). The tensile stresses were enhanced from 9.1±1.4 MPa in B_100_A_0_-M_6_ hydrogels to 40.1±5.3 MPa in B_95_A_5_-M_6_ ones and further increased to the largest value of 52.6±5.9 MPa in B_60_A_40_-M_6_ hydrogels (Figure S10). Meanwhile, the fracture elongations of the hydrogels containing alginate-Ca^2+^ were not compromised with the high stresses but also improved obviously (Figure 2A and S10). For instance, the strains were elongated from 4.1±0.6 in B_100_A_0_-M_6_ hydrogels to 6.1±0.2 % in B_95_A_5_-M_6_ ones (Figure S10). The Young's modulus was increased from 360.8±125.7 MPa in B_100_A_0_-M_6_ hydrogels to 848.3±85.8 MPa in B_95_A_5_-M_6_ ones, and the work of fracture did from 279.0±88.0 to 1339.2±175.3 KJ/m^3^ (Figure 2B). Along with the amount of alginate increased, Young's modulus of the hydrogels was increased and reached the highest value of 1671.0±141.5 MPa in B_40_A_60_-M_6_ hydrogels, and the work of fracture reached the highest value of 1690.9±161.9 KJ/m^3^ in B_80_A_20_-M_6_ hydrogels (Figure 2B). Additionally, the B_0_A_100_ hydrogel deformed during the mineralization process, and it became too brittle to perform a stress-strain test after mineralization for 6 days (Figure S11). The composite proportions in B_100_A_100-x_-M_6_ hydrogels were also determined. They all had similar component percentages of ~20 wt% organics, i.e. BC and alginate, ~30 wt% inorganics, and ~50 wt% water (Figure S12), which indicates alginate-Ca^2+^ played a critical role in the mechanical properties of the hydrogels.

The B_80_A_20_-M_6_ hydrogels that possessed both high stiffness and toughness were further studied. The hydrogels mineralized for different days (B_80_A_20_-M_y_) were measured for their mechanical properties. The initial hydrogels of B_80_A_20_-M_0_ showed a relatively large fracture strain (12.9±2.1 %) but very weak fracture stress (5.4±1.7 MPa, Figure 2C-D). When the mineralization time was prolonged, the fracture stresses of the hydrogels were gradually increased and reached a maximum of 51.8±1.4 MPa on the 7^th^ day, and the fracture strains were gradually reduced in compromise (Figure 2C-D). The Young's modulus of the mineralized hydrogels was increased along with the mineralization days and reached the highest value of 1640.0±150.0 MPa on the 8^th^ day (Figure 2E). The work of fracture of the mineralized hydrogels reached the highest value of 1608.4±144.5 KJ/m^3^ on the 6^th^ day and then decreased due to the reduced fracture strains (Figure 2E). The compositions of hydrogels mineralized for different days were further determined. The weight percentages of CaP quickly increased at the first three days (from 0.5 wt% on 0^th^ day to 28.4 wt% on the 3^rd^ day), and then slowly reached a maximum ratio of 31.6 wt% on the 8^th^ day (Figure 2F and S13). The fracture energies of the mineralized hydrogels were also increased along with the mineralization times and reached the highest value of 3012.7±306.4 J/m^2^ on the 6^th^ day (Figure 2G). Overall, B_80_A_20_-M_6_ hydrogels possessed the optimized mechanical properties of fracture stress of 47.9±2.4 MPa, Young's modulus of 1329.0±116.1 MPa, work of fracture of 1608.4±144.5 KJ/m^3^, and fracture energy of 3012.7±306.4 J/m^2^. A small piece of B_80_A_20_-M_6_ hydrogel (0.2 x 8 x 60 mm) could even lift a 2 kg weight (Figure S14).

To explore the mechanism of high mechanical properties, the stress-strain curves of B_100_A_0_-M_6_, B_80_A_20_-M_6_, and B_60_A_40_-M_6_ hydrogels were tested for one loading-unloading cycle at 1% strain. The noticeable hysteresis was observed in B_80_A_20_-M_6_ and B_60_A_40_-M_6_ hydrogels, while B_100_A_0_-M_6_ hydrogel showed negligible hysteresis (Figure 2H). The dissipated energies of B_80_A_20_-M_6_ and B_60_A_40_-M_6_ hydrogels were 16.3±7.6 KJ/m^3^ and 23.24±9.2 KJ/m^3^, respectively, and that of B_100_A_0_-M_6_ hydrogels was only 1.7±0.8 KJ/m^3^. The data suggest alginate-Ca^2+^ network efficiently dissipated energies in the hybrid hydrogels. Moreover, BC@CaP and alginate-Ca^2+^ formed a brick-and-mortar-like structure. When stretched, BC@CaP nanostructures were broken in a “tortuous fracture path” fashion, and the alginate-Ca^2+^ network was unzipped (Figure 2I). Since BC@CaP nanostructures and alginate-Ca^2+^ were interwoven, BC@CaP nanostructures might additionally enlarge the dissipating energy by amplifying the unzipping zoom of the alginate-Ca^2+^ network (Figure 2I). Taken together, the “tortuous fracture path” mechanism and the hysteresis by unzipping ionic networks should make a great contribution to the mechanical properties of the hydrogels.

### Characterization of B_80_A_20_-M_y_ hydrogels

**Figure 3:**
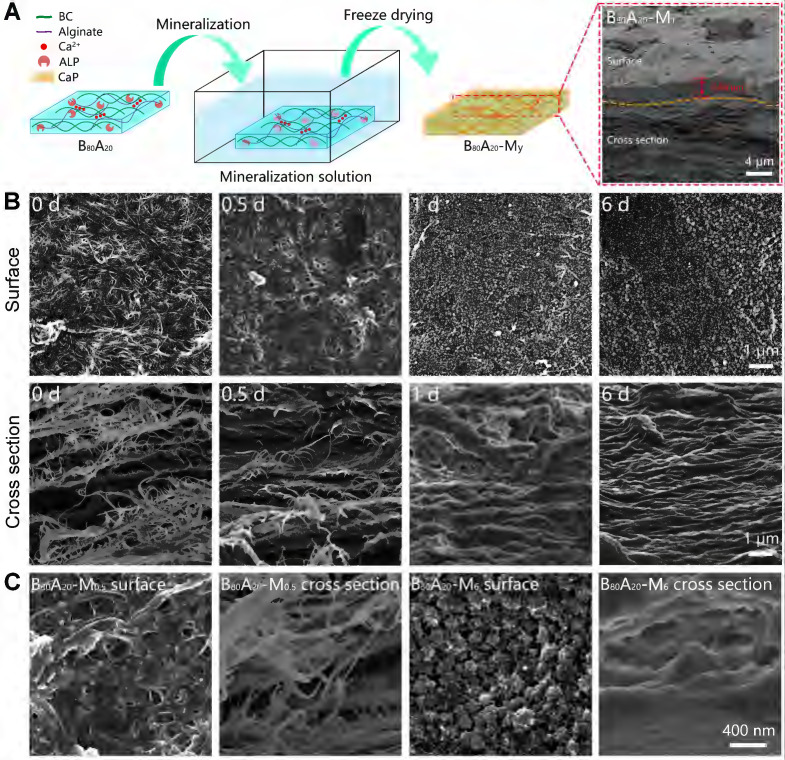
** Structural and compositional properties of mineralized hydrogels.** (**A**) Schematic illustration of the mineralization process of B_80_A_20_ hydrogel. Inset is the SEM image of B_80_A_20_-M_1_ hydrogel. (**B**) SEM images show the surface and cross-section profiles of B_80_A_20_ hydrogels mineralized for different days. (**C**) The amplified SEM images of B_80_A_20_-M_0.5_ and B_80_A_20_-M_6_ hydrogels.

**Figure 4:**
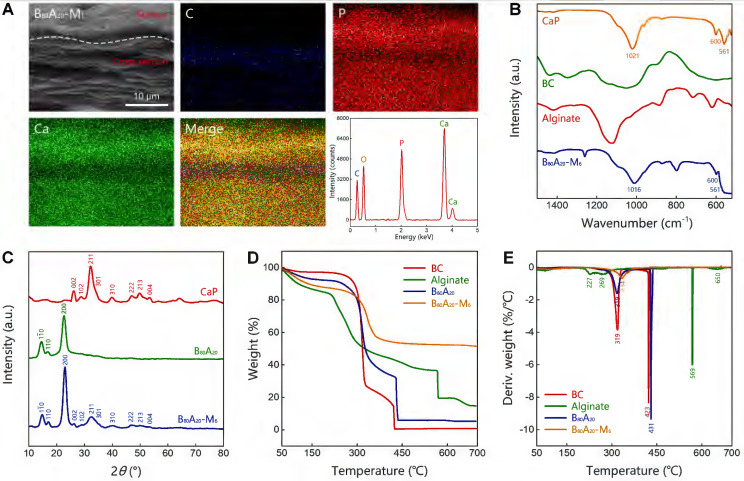
** Characterization of B_80_A_20_-M_6_ hydrogel.** (**A**) EDX element mapping of B_80_A_20_-M_1_ hydrogel (C stands for carbon element, P for phosphorus, and Ca for calcium). (**B**) FTIR spectra of B_80_A_20_-M_6_ hydrogel, BC, alginate, and CaP. (**C**) The X-ray diffraction spectra of B_80_A_20_ hydrogel, B_80_A_20_-M_6_ hydrogel, and CaP. CaP in b and c was produced via ALP-catalyzed deposition of CaGP in TEA buffer. (**D and E**) The thermogravimetric analysis and derivative thermogravimetric curves of B_80_A_20_, B_80_A_20_-M_6_ hydrogels, BC, and alginate.

The microstructures of the mineralized hydrogels were observed. B_80_A_20_ hydrogels were immersed in the mineralization solution for different days and then were lyophilized for scanning electron microscopy (SEM) observation (Figure 3A). A whole SEM view of B_80_A_20_-M_1_ hydrogel reveals that there were two distinct morphologies exited in the mineralized hydrogel, a compact layer on the surface and a series of lamellar structures below the surface (Figure 3A). The compact surface layer was ~2.8 µm thick (Figure 3A). The energy-dispersive X‐ray spectroscopy (EDX) element mapping determines that the compact surface layer was composed of CaP (denoted by Ca and P elements) but not organic components (denoted by C element, Figure 4A). However, the lamellar structures were indicated to have both organic components and inorganic CaP (Figure 4A). The EDX element mapping from the top view of the hydrogel confirms that the surface of B_80_A_20_-M_6_ hydrogels was majorly composed of CaP (Figure S15). During the mineralization process, CaGP diffused from the buffer solution into hydrogels and was preferentially decomposed at their interfaces, leading to the formation of such a heterostructure. Furthermore, the surface topography of B_80_A_20_-M_6_ hydrogel was observed by using atomic force microscopy. Its representative topography image reveals that the material had a flat surface (Figure S16). The arithmetic mean and the root means square surface roughness of B_80_A_20_-M_6_ hydrogel were only 1.33±0.25 and 2.47±0.32 nm, respectively. The friction coefficient of B_80_A_20_-M_6_ hydrogel was measured to be 0.37 (Figure S17), in which CaP nanoparticles should contribute to the friction coefficient. The microstructures of B_80_A_20_ hydrogel after mineralization for different days were shown in Figure 3B. In B_80_A_20_ hydrogel, BC nanofibers were observed (Figure 3B). On the surface, BC intertwined to form a compact network, while on the cross-section profile, they formed lamellar sheets with large intervals (Figure 3B). The unique structure was probably due to the hydrogel prepared via a blade casting method, and BC nanofibers tended to be arranged in the plane when the film was dried. After mineralization for 0.5 days, the hydrogel was deposited with CaP components on the surface and inside of the gel matrix (Figure 3B). The zoom-in images reveal that clusters of CaP nanoparticles were formed on the surface of hydrogel, while in the hydrogels (cross-section view) CaP was deposited on the surface of BC nanofibers to form a continuous coating (Figure 3C). After mineralization for 1 day, the surface of the hydrogel was covered by the high density of CaP nanoparticles, while the cross-section of the hydrogel became denser, and BC nanofibers were conjoined by CaP (Figure 3B). In this case, the CaP structure was quite different from that observed in the polymer matrix 33. The polymer hydrogels could not offer nucleating points for CaP. As a result, CaP nanoparticles were formed at the intervals of a polymer network 33. In comparison, BC nanofibers provided the sites for CaP nucleation and deposition due to the negative charges on the surface (Figure 1E and S1). As a result, CaP nanoparticles were constantly deposited on the surface of BC nanofibers, and finally, a high density of BC@CaP nanostructures was formed on the 6^th^ day (Figure 3B). The zoom-in images reveal that flower-like CaP nanoparticles were connected with internal bridges on the surface of B_80_A_20_-M_6_ hydrogel, and in the cross-section, BC@CaP nanofibers had fused to form a compact structure (Figure 3C).

The Fourier transform infrared spectroscopy (FTIR) analysis of B_80_A_20_-M_6_ hydrogel and the individual components including CaP from ALP-catalyzed hydrolysis of CaGP, BC nanofibers, alginate was conducted (Figure 4B and S18). B_80_A_20_-M_6_ hydrogel possessed the characteristic peaks of all components. Especially, CaP power represented one sharp peak at 1021 cm^-1^ for asymmetric PO_4_^3-^ stretching vibrations, and two peaks at 600 and 561 cm^-1^ for asymmetric PO_4_^3-^ deformation vibrations, which indicates the mineralized CaP was made of hydroxyapatite 46. B_80_A_20_-M_6_ hydrogel contained the typical peaks of hydroxyapatite, suggesting CaP deposited in the hydrogel should be hydroxyapatite. The X-ray diffraction spectra of CaP, B_80_A_20_ hydrogel, and B_80_A_20_-M_6_ one were also measured. The CaP powder showed multiple diffraction peaks at 26.1°, 28.5°, 32.3°, 33.3°, 34.1°, 39.7°, 46.9°, 48.2°, 50.0°, and 53.4° (Figure 4C), which were corresponding to the typical lattice plane of hydroxyapatite (002) m, (102) m, (211) m, (112) m, (301) m, (310) m, (222) m, (312) m, (213) m and (004) m 47. B_80_A_20_ hydrogel showed the typical diffraction peaks of BC at 14.7°, 16.7°, and 22.7° (Figure 4C) 48. B_80_A_20_-M_6_ hydrogel contained both the typical diffraction peaks of BC and hydroxyapatite (Figure 4C). Taken together, we confirmed that CaP in the hydrogel was hydroxyapatite. The dried B_80_A_20_ and B_80_A_20_-M_6_ hydrogels were further assessed by thermogravimetric analysis. The thermogravimetric curves show that the dried B_80_A_20_ hydrogel was majorly pyrolyzed at a temperature ranging from 250 to 435 °C, and the dried B_80_A_20_-M_6_ hydrogel did between 280 to 365 °C (Figure 4D). The dried B_80_A_20_ hydrogel was completely pyrolyzed due to its organic composition, while the dried B_80_A_20_-M_6_ hydrogel was pyrolyzed only 47.6 wt% (Figure 4D). The non-pyrolyzed component should be CaP (52.4 wt%, Figure 4D). The derivative thermogravimetric curves reveal that the maximum weight loss rate for dried B_80_A_20_ hydrogel was 430.6 °C and that for dried B_80_A_20_-M_6_ hydrogel was 334.1 °C (Figure 4E).

### *In situ* mineralization in the cartilage defect

**Figure 5:**
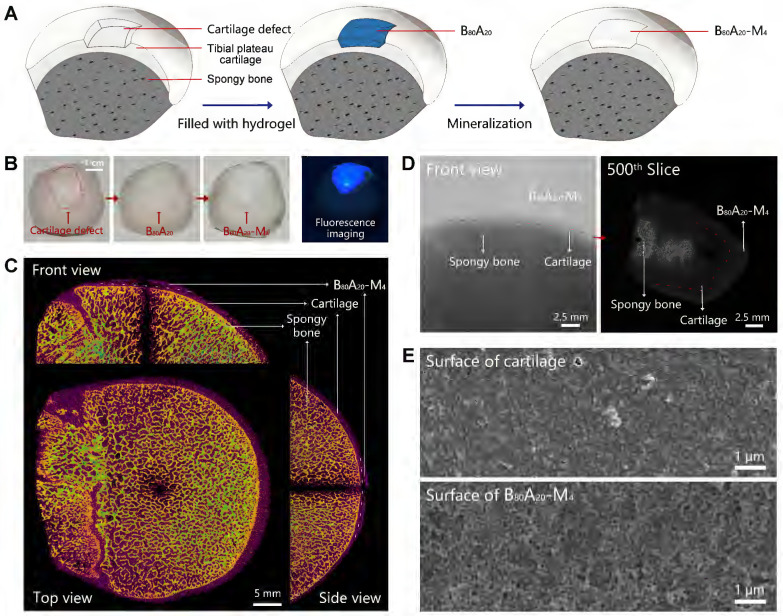
***In-situ* mineralization in the cartilage defect.** (**A**) The scheme shows the mineralization process in a man-made articular cartilage defect *ex vivo*. (**B**) Photographs of the cartilage defect before and after being filled with B_80_A_20_-M_4_ hydrogel (labeled with calcofluor white stain). (**C**) The orthographic views of the cartilage defect filled with B_80_A_20_-M_4_ hydrogel (front, top, and side view). (**D**) The representative micro-CT images of the cartilage defect filled with B_80_A_20_-M_4_ hydrogel (front view and the 500^th^ slice from the top down). The red line represents the boundary between B_80_A_20_-M_4_ hydrogel and natural cartilage. (**E**) SEM images of the surface of natural cartilage and B_80_A_20_-M_4_ hydrogel in the defect.

Cartilage defects can be divided into three types includes partial-thickness, full-thickness, and osteochondral defects. In the full-thickness and osteochondral defects, subchondral bone also needs to be repaired except for cartilage. Here, we employed B_80_A_20_-M_4_ hydrogel as a replacement material to fix the subchondral bone damage. The assay was carried out via *in situ* mineralization of CaP in the hydrogel in a man-made articular cartilage defect model* ex vivo* (Figure 5A). ALP is an enzyme that wildly exists in body tissue 49. In bone tissue, ALPs regulate bone mineralization via hydrolyze pyrophosphate and also supply inorganic phosphate for the synthesis of hydroxyapatite via hydrolyze pyrophosphate and organic phosphomonoesters 50. Therefore, ALP-catalyzed CaP mineralization in B_80_A_20_-M_4_ hydrogel might mimic the natural synthesis of CaP in bone tissues. Moreover, a partial-thickness cartilage defect was created instead of full-thickness or osteochondral defects, and thus the mineralized hydrogel directly adhered to the cartilage tissue in the defect, which facilitated the characterization of the mineralized hydrogel and its binding to the natural cartilage. The defect was filled with B_80_A_20_ hydrogel and then fed with the mineralization solution (Figure 5A). After repair, the defect region was deposited with CaP in the gel matrix, where the mineralized hydrogel perfectly integrated with the natural cartilage tissues at the boundary (Figure 5B). A fluorescent agent of calcofluor white stain was embedded in B_80_A_20_ hydrogel to indicate the location of repaired area (Figure 5B). Furtherly, the bone was imaged by micro-computerized tomography (micro-CT). The CT image reveals that the repaired region had a similar microstructure and density compared with the natural bone tissue (Figure 5C). The section CT further reveals that B_80_A_20_-M_4_ hydrogel was tightly incorporated with natural cartilage tissue (Figure 5D). The SEM images reveal that the *in situ* formed B_80_A_20_-M_4_ hydrogel was composed of BC nanofibers and CaP nanoparticles, while the natural cartilage showed a dense structure that was probably the extracellular matrix (Figure 5E and S19). The assay demonstrates that the hydrogel could be used for subchondral bone repair in the cartilage defect.

## Conclusion

In summary, we have prepared a hydrogel with ultrahigh strength, stiffness, and toughness. In the hydrogel, BC and alginate-Ca^2+^ formed a crosslinked double network, and then ALPs induced CaP deposition in the organic matrix. BC nanofibers were used as the main component of the gel matrix instead of molecular polymers, which themselves were strong enough to reinforce the hydrogel, and also templated the deposition of CaP to form BC@CaP nanostructures. Then, BC@CaP and alginate-Ca^2+^ formed a brick-and-mortar-like structure. When stretched, the “tortuous fracture path” breaking among BC@CaP nanostructures and the hysteresis by unzipping the alginate-Ca^2+^ network worked synergistically, resulting in great improvement over the mechanical properties of the hydrogels. The best hydrogel possessed ultrahigh fracture stress of 48 MPa, Young's modulus of 1329 MPa, and fracture energy of 3013 J/m^2^. All three properties are superior to the parameters of most of the reported synthetic hydrogels with high mechanical behaviors. Moreover, the successful case for the* ex vivo* repair of subchondral bone defect suggests that this hydrogel has great promise in bone tissue engineering.

## Methods

### Materials

BC membrane was obtained from Hainan Yeguo Foods Co., Ltd (Hainan, China). Sodium alginate, TEA, glutaraldehyde (aqueous solution, 50 wt %), sodium hydroxide, and calcium chloride were purchased from Macklin Biochemical Co., Ltd (Shanghai, China). ALPs extracted from the calf intestine were obtained from AppliChem GmbH (Darmstadt, Germany). CaGP, 4-nitrophenyl phosphate disodium salt hexahydrate, and p-nitrophenol were bought from Alfa Aesar Chemicals Co., Ltd (Shanghai, China). Hydrochloric acid and dimethyl sulfoxide were obtained from Sinopharm Chemical Reagent Co., Ltd (Shanghai, China).

### BC slurry preparation

BC membrane was smashed into small pieces using a blender (MQ5025, Braun, Germany), and then treated by a high-pressure homogenizer (1000 bar, AH-PILOT 2018, ATS Engineering Limited, China) three times.

### Synthesis of PGL

PGL was synthesized based on a reported method 51. 20 mL glutaraldehyde aqueous solution (50 wt %) was added in a mix of 15 mL deionized (DI) water and 5 mL dimethyl sulfoxide. The pH value of the solution was adjusted to 10.5 by adding sodium hydroxide (1 M). After stirring for 30 min, the solution was neutralized with hydrochloric acid (1 M), and then diluted ten times with DI water. The PGL solution was stored at 4 °C for use.

### Preparation of ALP/PGL nanoparticles

The ALP/PGL nanoparticles were prepared via a reported method 52. 0.1 mL of freshly-prepared ALPs (10 mg/mL) in DI water was mixed with different amounts of PGL solution (23.8 mg/mL, 0, 4.2, 12.6, 25.2, 50.4, and 105 μL) using a vortex mixer. After standing for 5 min, ALP/PGL nanoparticles were obtained and stored at 4 °C for use.

### Enzyme activity of ALP/PGL nanoparticles

The enzyme activities of ALP/PGL nanoparticles were determined via a standard method. Before the assay, the standard curve of p-nitrophenol (in 0.2 M TEA buffer, pH = 9.8) was determined by measuring its absorption (405 nm) at different concentrations using an ultraviolet spectrophotometer (Cary 60, Agilent Technologies, USA). To determine the enzyme activity, 1 mL of ALP/PGL suspension (5 µg/mL for ALPs, in 0.2 M TEA buffer, pH = 9.8) was added in a quartz cuvette, which was held with 1 mL of 4-nitrophenyl phosphate disodium salt hexahydrate solution (0.1 M, in 0.2 M TEA buffer, pH = 9.8). The absorbance of the solution at 405 nm was recorded by the ultraviolet spectrophotometer over time. Finally, the specific activity (U/mg) of ALP/PGL was calculated according to the definition of active unit (the amount of enzyme required to convert 1 μM of the substrate within 1 min is defined as an international unit of enzyme activity).

### Preparation of B_x_/A_100-x_ hydrogels

In a typical preparation of B_80_A_20_ hydrogel, 16 mL of BC aqueous suspension (0.4 wt%) and 4 mL of sodium alginate aqueous solution (0.4 wt%) were mixed, and then 312.6 μL of ALP/PGL suspension (10.6 mg/mL) was added. After homogenously mixing with a vortex mixer, the bubbles trapped in the solution were removed by ultrasonic treatment in an ice bath. After that, the solution was poured into a homemade rectangular mold (4 x 8 x 1 cm) made of polymethyl methacrylate plate. The sample was dried in a fume and then immersed in calcium chloride solution (5 mg/mL) for 30 min. B_x_A_100-x_ hydrogels with different weight ratios of BC and sodium alginate were prepared in the same way.

### Mineralization of B_x_/A_100-x_ hydrogels

In a typical case, B_80_A_20_ hydrogel was immersed in 80 mL of mineralization solution at room temperature. The mineralization solution was replaced every day. After incubation for 6 days, B_80_A_20_-M_6_ hydrogel was obtained. The hydrogel was washed with DI water three times and then stored in DI water at 4 °C. The mineralization solution was prepared by dissolving 5 g of CaGP into 1 L of 0.2 M TEA buffer, and the pH value was adjusted to 9.8 by adding 5 M hydrochloric acid. B_x_A_100-x_-M_y_ hydrogels were prepared in the same way.

### Characterization

The SEM images and EDX mapping of hydrogels were obtained by using a scanning electron microscope (S-4800, Hitachi, Japan) coupled with an energy-dispersive X‐ray spectroscope. The FTIR spectra were collected by using a Fourier transform infrared spectroscope (Nicolet iS50, Thermo Fisher Scientific, America). The thermogravimetric analysis was conducted by using a thermogravimetric analyzer (TGA4000, PerkinElmer, America). The X-ray diffraction spectra were determined by using an X-ray diffractometer (Smartlab SE, Rigaku, Japan) within a 2*θ* range from 5 to 140°. The crystallinity Index of BC in hydrogels was calculated according to the reported method.^35^ The hydrodynamic size and zeta potential of materials were measured by using a dynamic light scattering (Zetasizer Nano ZS90, Malvern, UK).

### Composition of B_x_A_100-x_-M_y_ hydrogel

The composition of B_x_A_100-x_-M_y_ hydrogel was determined by a gravimetric method. The dry weight of B_x_A_100-x_ hydrogel (m_1_), and the dry weight (m_2_) and wet weights (m_3_) of the mineralized B_x_A_100-x_-M_y_ hydrogel were measured. The percentages of the contents were calculated as follows:


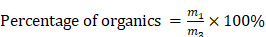

(1)


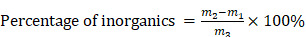

(2)


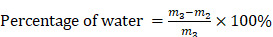

(3)

### Mechanical properties of hydrogels

The mechanical properties of B_x_A_100-x_-M_y_ hydrogel were evaluated by using an electronic tensile testing machine (HY-0580, Shanghai Hengyi Testing Instruments Co., Ltd, China). The hydrogels in 0.05-0.3 mm thickness were cut into the testing samples with a strip shape of 5 mm width x 25 mm length by using a surgical blade. The samples were fixed by using clamps with an initial distance of 16 mm and then were stretched at a speed of 20 mm/min to obtain the stress-strain curves. The strain (%) was regarded as the length change related to the initial length of the sample. The stress (MPa) was calculated by dividing the force by the initial cross-sectional area of the samples. The Young's modulus (MPa) was determined from the slope of the initial linear region of the stress-strain curves. The work of fracture (KJ/m^3^) was determined by the area below the stress-strain curves. The dissipated energy (KJ/m^3^) was estimated by the area between the loading-unloading curves.

The fracture energy (J/m^2^) of B_x_A_100-x_-M_y_ hydrogel was estimated with a standard method introduced by Rivlin and Thomas 53. Briefly, two rectangular samples (8 mm width x 25 mm length) separated from the same hydrogel were used for measurement. One was intact, and the other was cut with a 4 mm notch using a surgical blade. The two samples were stretched at a speed of 20 mm/min to obtain the stress-strain curves. The corresponding fracture energy (*Γ*) of the unnotched samples was calculated as follows:


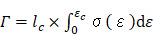

(4)

*Γ*: The fracture energy (J/m^2^)

*ε_c_*: The fracture strain of the notched samples (%)

σ: The stress of the unnotched samples (MPa)

*l_c_*: The initial length of the samples (mm)

### Surface roughness analysis

The surface topography of B_80_A_20_-M_6_ hydrogel was observed by using atomic force microscopy (Dimension Icon, BRUKER, America) and the arithmetic mean and the root means square surface roughness were analyzed by a professional analysis software *NanoScope Analysis 3.0*.

### Friction testing

The friction coefficient of B_80_A_20_-M_6_ hydrogel was analyzed by using high-speed reciprocating friction and wear tester (MDW-02G, Jinan Yihua Tribology Testing Technology Co., Ltd, China) operated in reciprocating sliding mode. To simulate the frictional environment *in vivo*, the entire testing process was performed in PBS. In all of the tests, the sliding speed was 0.5 mm/s, the length of the wear track was 10 mm, and the normal load was 10 N, resulting in an average contact pressure of 0.1 MPa. In addition, the glass disk was ultrasonically cleaned after each test to ensure a fresh surface. The friction coefficient was calculated as follows:



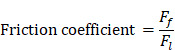




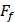
: The friction force (N)


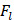
: The normal load (N).

### *Ex-vivo* repair of subchondral bone in the cartilage defect

The *Ex-vivo* model of cartilage defect was established on the tibial plateau cartilage of pork bone, which was bought from the supermarket. The defect was made by excising the cartilage section using a surgical blade. The size of the defect was approximately 1 cm width × 1.5 cm length × 0.5 mm depth. The mixture of BC, sodium alginate, and ALP/PGL for B_80_A_20_ hydrogel was prepared as above, and 50 μL calcofluor white stain was added. It was filled into the defect and then infiltrated by 2 mL of 5 mg/mL calcium chloride solution for 30 min. After that, the defect covered with hydrogel was flushed with DI water three times. Finally, 2 mL of mineralization solution was added at the defect location. The mineralization solution was changed twice a day, and the sample was mineralized for 4 days.

### Micro-CT analysis

The micro-CT analysis of bone was performed by using an x-ray microtomography (SKYSCAN 1272, BRUKER, Germany). The samples were scanned at a source voltage of 60.0 kV and a source current of 100 μA for 70 min with a camera pixel size resolution of 22 μm. The region of interest was located from 1.232 mm (the 56^th^ slice) to 16.874 mm (the 767^th^ slice) where the bottom of the tibial plateau cartilage was defined as 0 mm. 3D reconstruction images were produced with CTVOL software (version 1.1.10, Bruker micro-CT).

## Supplementary Material

Supplementary figures.

## Abbreviations

BC: bacterial cellulose
CaP: calcium phosphate
TEA: triethanolamine
ALPs: alkaline phosphatases
CaGP: calcium glycerophosphate
PGL: poly-glutaraldehyde
DI: deionized
SEM: scanning electron microscopy
FTIR: Fourier transform infrared
EDX: energy-dispersive X‐ray spectroscope
micro-CT: micro-computerized tomography
