# Supplementary material for: Enzymatically-mineralized double-network hydrogels with ultrahigh mechanical strength, toughness, and stiffness
Source: Theranostics. 2023 Jan 1;13(2):673–84. doi: 10.7150/thno.77417 (PMC9830447; doi:10.7150/thno.77417)
Supplement: Supplementary file 1 — Supplementary figures. [file thnov13p0673s1.pdf]

Supplementary Material

Enzymatically-mineralized double-network hydrogels with ultrahigh mechanical strength, toughness, and stiffness

Li Wang [2], Wei Zhao [1], Yining Zhao [2], Wei Li [1,*], Guodong Wang [1,*] and Qiang Zhang [1,2,*]

[1]Department of Stomatology, Changzheng Hospital, Naval Medical University, Shanghai, 200003, P. R. China

[2]Shanghai Key Laboratory of Regulatory Biology, School of Life Sciences, East China Normal University, Shanghai, 200241, P.R. China

*Correspondence should be addressed to Q. Z. (E-mail: qzhang@bio.ecnu.edu.cn); G. W. (E-mail: wangguodong@smmu.edu.cn); W. L. (E-mail: li_wei_sh@hotmail. com)

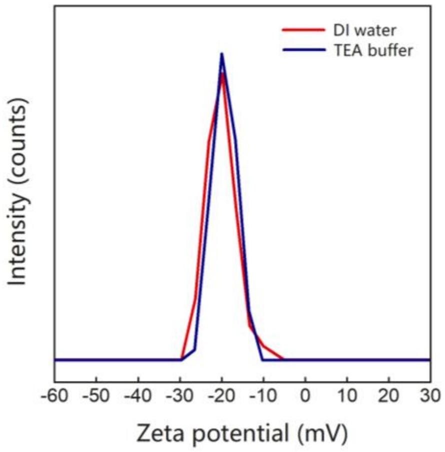

Figure S1. The zeta potential of BC nanofibers in DI water (pH = 7.0) and TEA buffer (pH = 9.8).

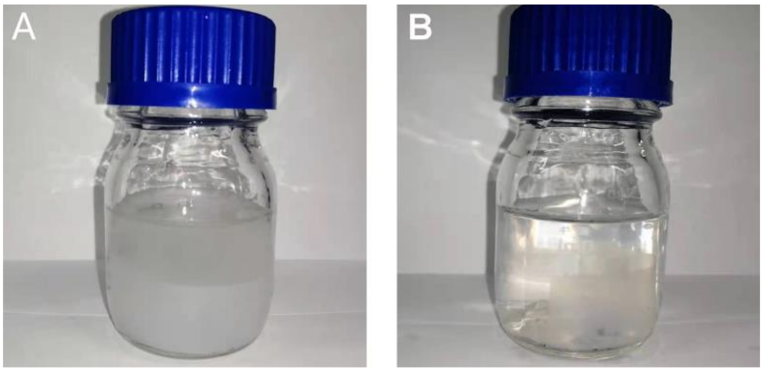

Figure S2. (**A**) $B_{80}A_{20}$ hydrogel containing ALPs was mineralized in a mineralization solution (0.20 M TEA buffer containing 5 g/L CaGP, pH = 9.8). The solution became cloudy rapidly after mineralization for 1-2 h because the small ALPs (~ 2.8 nm) leaked out of the hydrogel. (**B**) $B_{80}A_{20}$ hydrogel containing ALP/PGL nanoparticles with a weight ratio of 10:1 was mineralized in a mineralization solution. The solution was kept clear during the mineralization process, which indicates ALP/PGL nanoparticles (108 nm) were trapped in the hydrogel.

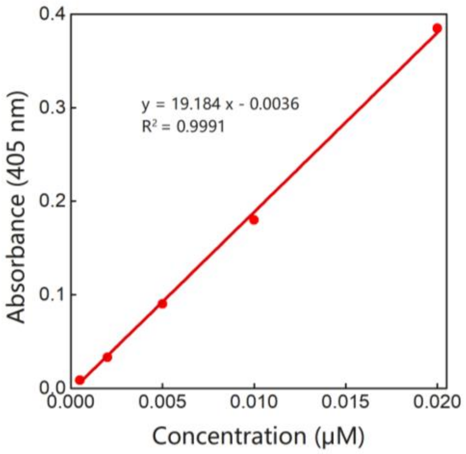

Figure S3. The calibration curve of p-nitrophenol. The absorptions of p-nitrophenol of different concentrations at 405 nm were determined by using an ultraviolet spectrophotometer (Cary 60, Agilent Technologies, USA). The enzyme activity of ALP was determined according to the standard curves of p-nitrophenol, as ALP catalyzed the decomposition of p-nitrophenol phosphonate to release p-nitrophenol.

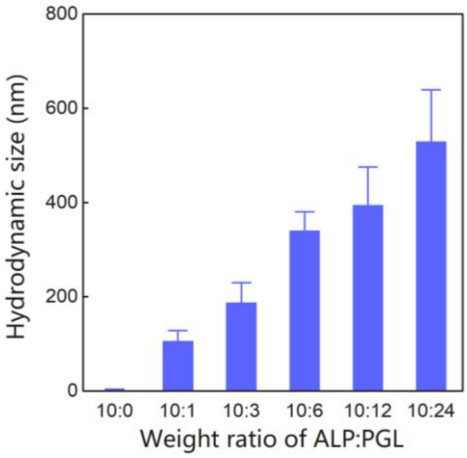

Figure S4. Hydrodynamic sizes of ALP/PGL nanoparticles. ALP/PGL nanoparticles with different weight ratios of ALP: PGL (10:0, 10:1, 10:3, 10:6, 10:12, and 10:24) were measured by dynamic light scattering (Zetasizer Nano ZS90, Malvern, UK).

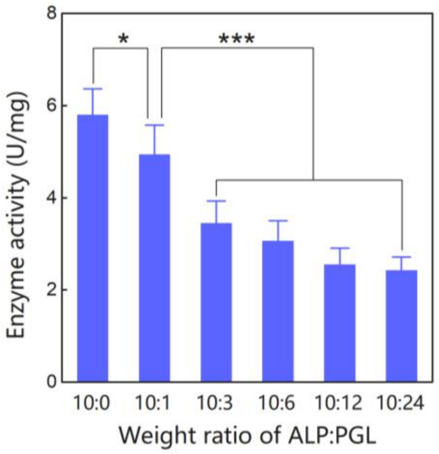

Figure S5. The enzyme activities of ALP/PGL nanoparticles with different weight ratios of ALP: PGL (10:0, 10:1, 10:3, 10:6, 10:12, and, 10:24) were measured by an ultraviolet spectrophotometer (Cary 60, Agilent Technologies, USA). The ALP/PGL nanoparticles with a weight ratio of 10 :1 showed a high enzymatic activity of 4.94 U/mg.

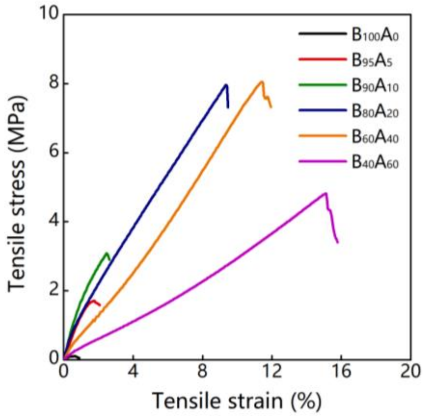

Figure S6. The stress-strain curves of B_xA_{100-x} hydrogels with different proportions of BC and alginate.

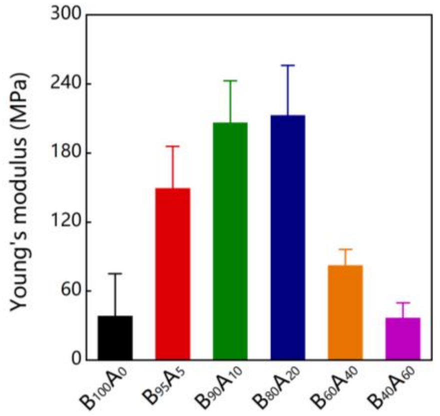

Figure S7. The Young's modulus of B_xA_{100-x} hydrogels with different proportions of BC and alginate (n=5).

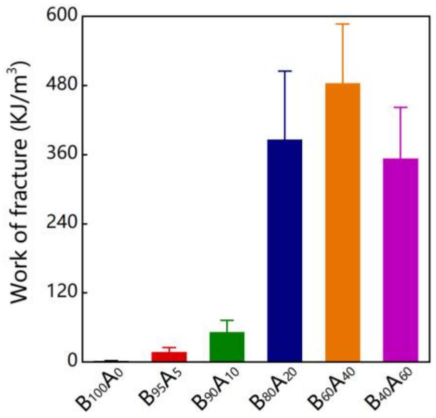

Figure S8. The work of fracture of B_xA_{100-x} hydrogels with different proportions of BC and alginate (n=5).

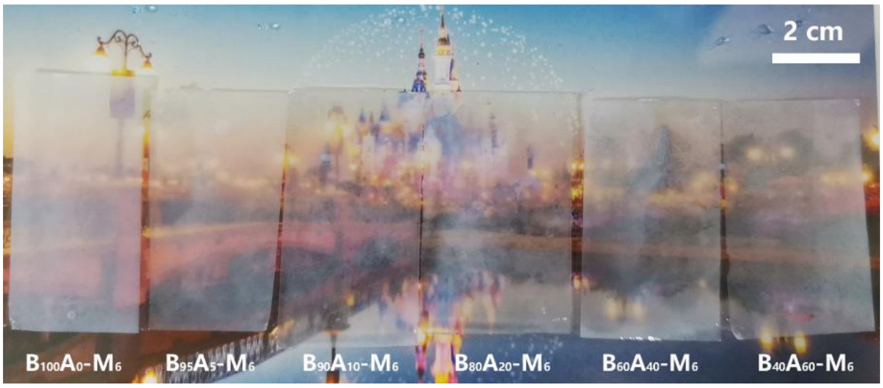

Figure S9. Photographs of B_xA_{100-x}-M_6 hydrogels with different proportions of BC and alginate.

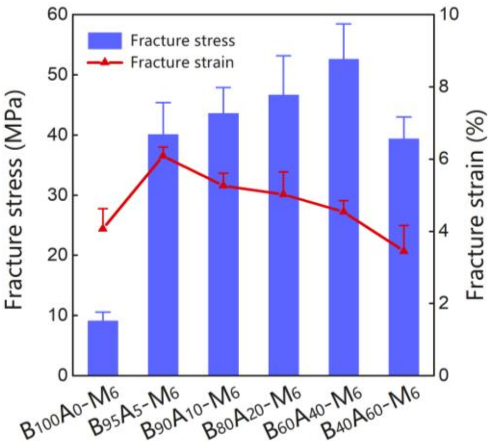

Figure S10. The fracture stress and fracture strain of B_xA_{100-x}-M_6 hydrogels with different proportions of BC and alginate (n=5).

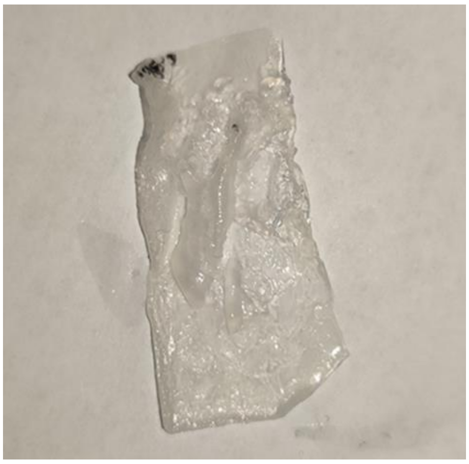

Figure S11. Photograph of B_0A_{100}-M_6 hydrogel. The hydrogel was wrinkled and brittle.

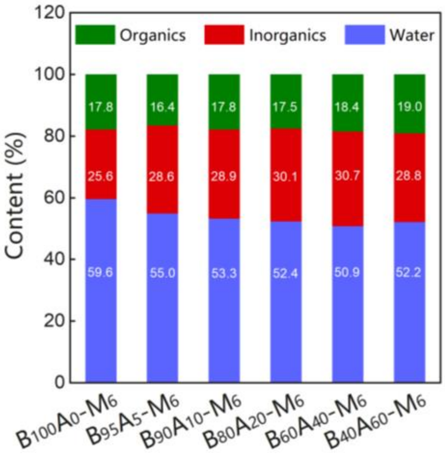

Figure S12. The proportions of organic components, inorganic ones, and water in different B_xA_{100-x}-M_6 hydrogels (n=5).

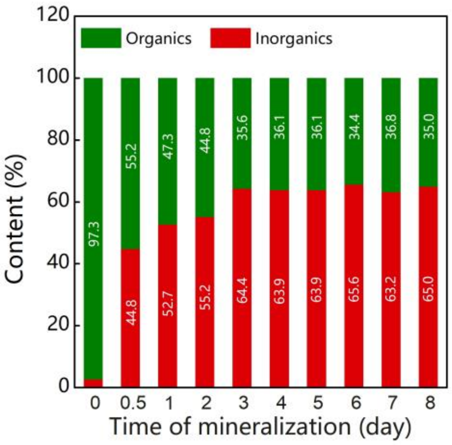

Figure S13. The contents of dried $B_{80}A_{20}$-M_y hydrogels after mineralization for different days (n=5).

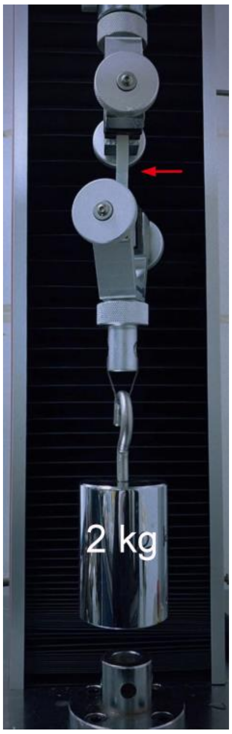

Figure S14. A rectangular sample of $B_{80}A_{20}$-M_6 hydrogel (0.2 x 8 x 60 mm) lifted 2 kg weights.

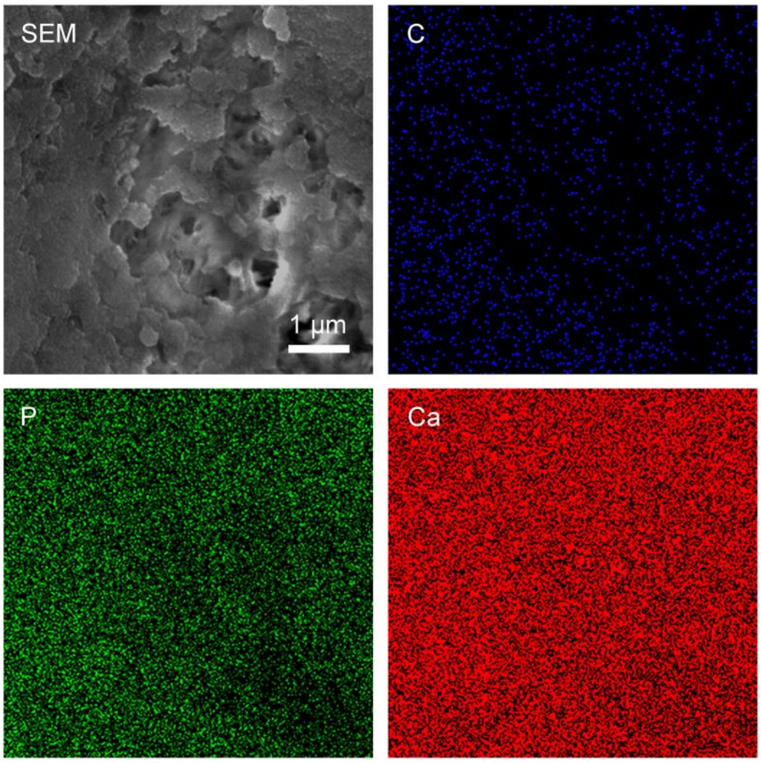

Figure S15. SEM image and EDX element mapping for the surface of $B_{80}A_{20}$-M_6 hydrogel.

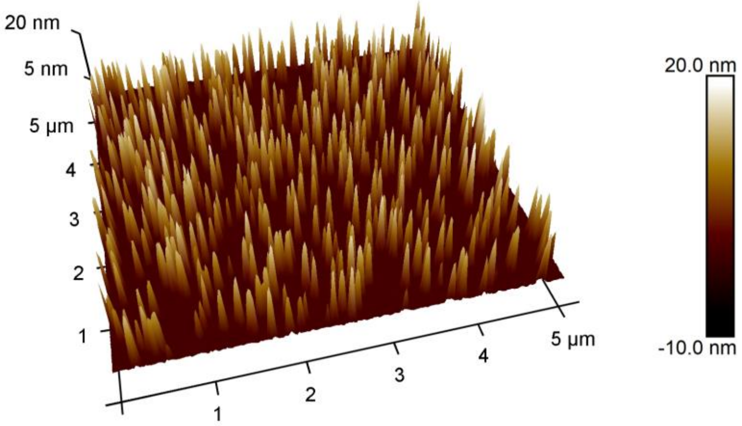

Figure S16. Topography image of $B_{80}A_{20}$-M_6 hydrogel.

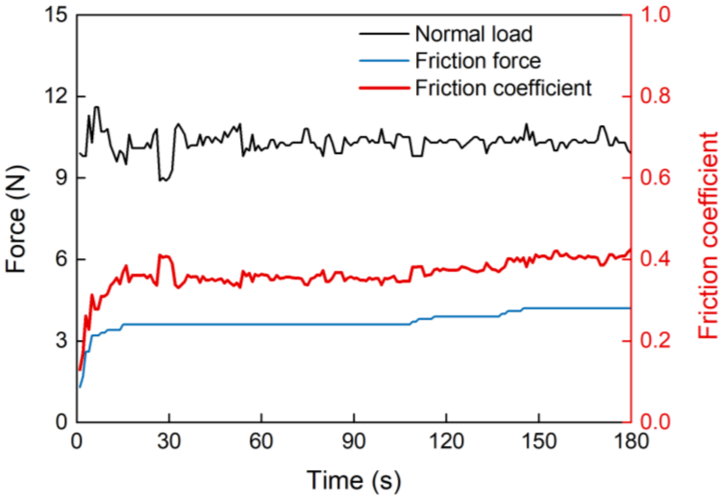

Figure S17. The normal load, friction force, and friction coefficient-time curves of $B_{80}A_{20}$-M_6 hydrogel.

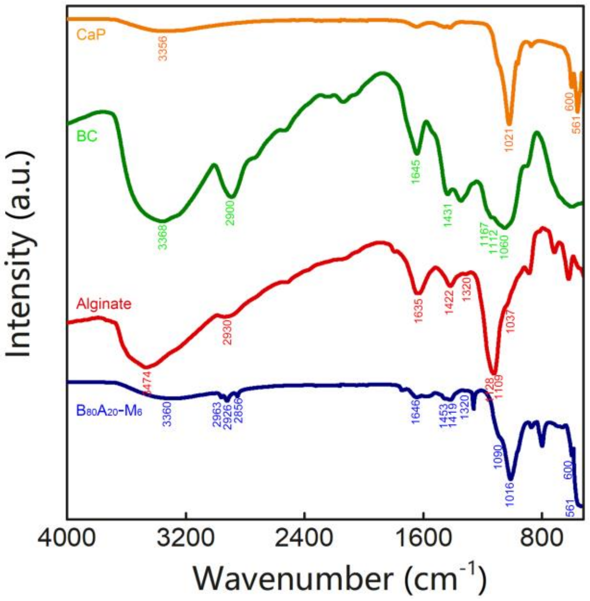

Figure S18. FTIR spectra of BCs, alginate, $B_{80}A_{20}$-M_6 hydrogel, and CaP.

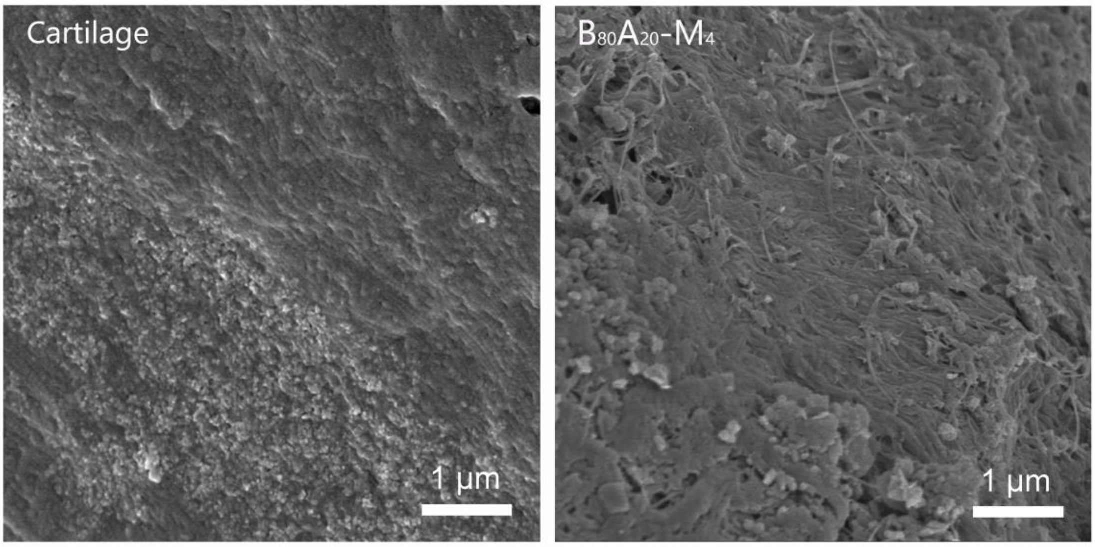

Figure S19. SEM images of the cross-section of natural cartilage and $B_{80}A_{20}$-M_4 hydrogel repaired area.
